# Evaluation of the cell-free DNA integrity index as a liquid biopsy marker to differentiate hepatocellular carcinoma from chronic liver disease

**DOI:** 10.3389/fmolb.2022.1024193

**Published:** 2022-11-22

**Authors:** Sonu Kumar, Neeti Nadda, Shashi Paul, Shivanand Gamanagatti, Nihar Ranjan Dash, Perumal Vanamail, Anoop Saraya, Shalimar, Baibaswata Nayak

**Affiliations:** ^1^ Department of Gastroenterology, All India Institute of Medical Sciences, New Delhi, India; ^2^ Department of Radiodiagnosis, All India Institute of Medical Sciences, New Delhi, India; ^3^ Department of Gastrointestinal Surgery, All India Institute of Medical Sciences, New Delhi, India; ^4^ Department of Biostatistics in Obstetrics and Gynaecology, All India Institute of Medical Sciences, New Delhi, India; ^5^ Trichy SRM Medical College Hospital & Research Centre, Tiruchirappalli, Tamil Nadu, India

**Keywords:** liquid biopsy, circulating free DNA, circulating tumor DNA, hepatocellular carcinoma, chronic liver disease, DNA integrity index

## Abstract

**Background:** Hepatocellular carcinoma (HCC) occurs in the majority of patients with underlying chronic liver disease (CLD) of viral and non-viral etiologies, which requires screening for early HCC diagnosis. Liquid biopsy holds great promise now for early detection, prognosis, and assessment of response to cancer therapy. Cell-free DNA (cfDNA) as a liquid biopsy marker can be easily detected by a real-time quantitative PCR (RT-qPCR) assay for a change in its concentration, integrity, and fragmentation in cancer.

**Methods:** Patients with HCC (n = 100), CLD (n = 100), and healthy (n = 30) controls were included in the study. The cfDNA was isolated from serum and real-time quantitative PCR (RT-qPCR) was carried out using primer pairs for large (>205 bp) and small (110 bp) fragments of repetitive elements (ALU and LINE1) and housekeeping genes (β-Actin and GAPDH). Total cfDNA concentrations and integrity index were determined by the absolute quantitation method (L/S ratio or cfDII-integrity). The cfDII as a measure of fragmentation was determined by comparative Ct (^2–ΔΔCt^) method of relative quantification (cfDII-fragmentation). Using a receiver operating characteristic (ROC) curve, cfDII-integrity and cfDII-fragmentation were used to differentiate HCC from CLD patients or healthy controls.

**Results:** The total cfDNA concentrations in the sera of HCC (244 ng/ml) patients were significantly higher than those of CLD (33 ng/ml) patients and healthy (16.88 ng/ml) controls. HCC patients have shown poor DNA integrity or excess cfDNA fragmentation than CLD patients and healthy controls. The cfDII-integrity of GAPDH and ALU fragment significantly differentiate HCC from CLD at AUROC 0.72 and 0.67, respectively. The cfDII-fragmentation following normalization with cfDNA of healthy control has shown significant differential capabilities of HCC from CLD at AUROC 0.67 using GAPDH and 0.68 using the ALU element. The ROC curve of LINE1 and β-actin cfDII was not found significant for any of the above methods. The cfDII-fragmentation trend in HCC patients of different etiologies was similar indicating increased cfDNA fragmentation irrespective of its etiology.

**Conclusion:** The cfDII measuring both DNA integrity (L/S ratio) and fragmentation of the Alu and GAPDH genes can differentiate HCC from CLD patients and healthy individuals.

## 1 Introduction

Hepatocellular carcinoma (HCC) accounts for approximately 90% of primary liver cancers (Llovet et al., 2021). As per Global Cancer Observatory (GLOBOCAN 2020) data, the liver cancer incidence rate in males is fifth, and in females, it is ninth among all forms of cancer. Of all the cancer-related deaths, the liver cancer-related mortality rate was second for males and sixth for females worldwide in 2020 (Sung et al., 2021). Primary liver cancer and liver metastasis account for 6.8 deaths per 100,000 people in India (Dikshit et al., 2012). HCC predominantly develops in patients with underlying chronic liver disease or cirrhosis (Valery et al., 2018). Chronic liver disease (CLD) is a progressive deterioration of liver functions for more than 6 months and can progress from liver inflammation to fibrosis, cirrhosis, HCC, and end-stage liver failure (Sharma and Nagalli, 2021). Chronic hepatitis B and C (CHB and CHC) are the major causes of HCC in the developing world, whereas non-alcoholic steatohepatitis (NASH) is the growing cause of HCC worldwide. The majority of HCC patients are diagnosed at an advanced stage and found unsuitable for curative treatments such as liver resection and liver transplantation (Yuen et al., 2000; Bruix and Llovet, 2002). Currently, imaging-based techniques (CT/MRI) are used for diagnosis, requiring infrastructure and a long wait period. Imaging-based techniques also do not give much insight into the molecular basis of HCC. Liquid biopsy-based biomarkers such as cell-free DNA (cfDNA), circulating tumor DNA (ctDNA), circulating tumor cells (CTCs), cancer stem cells (CSC), microRNA, and exosomes have recently been shown to be of importance in detection, prognosis, and prediction of response to cancer treatment. Recently, we have shown the role of microRNA as a liquid biopsy marker predicting response to locoregional therapy in HCC (Nadda et al., 2020). The advantages of liquid biopsies are the ease of detection and the non-invasive technique. This may have the potential to be a diagnostic and screening modality in the future (Qu et al., 2019).

Several Liquid biopsy-based tests are now approved by U.S. Food and Drug Administration (FDA) in clinical practice for non-small cell lung cancer (NSCLC) and colorectal cancer (CRC). The ctDNA-based test first to be approved by FDA is for the detection of EGFR mutations in NSCLC patients to start treatment with EGFR-TKIs (Rijavec et al., 2019; Abdayem and Planchard, 2021). Later, FDA also approved other broad NGS-based ctDNA tests such as Guardant360 and FoundationOne Liquid CDx to determine targeted therapies or chemoresistance for solid tumors (Rolfo et al., 2021). Detection of SEPT9 gene aberrant methylation (EpiProColon or mSEPT9 methylation test) for CRC screening is also another FDA-approved single gene related cfDNA-based test (Song et al., 2017). Now multimodal liquid biopsy-based test (LUNAR-2 or Shield Test) is on trial and showing promising results for early detection of CRC. This test includes ctDNA assessment of somatic mutations, tumor-derived methylation, and cfDNA fragmentations (Broccard et al., 2022). The ctDNAs are small fraction of cfDNA that mainly derived from primary tumors, metastatic tumors, and CTCs. The ctDNA carries entire tumor genetic information whereas there is spatial genetic heterogeneity in tumor tissue biopsy, Analysis of ctDNA gives a detail overview of the genomic landscape of tumour (Zhao et al., 2021).

The cfDNAs are originated both from tumor and extra-tumoral normal cells. The proportions of ctDNA in the total cfDNA greatly vary between <1% and >40%, depending upon clinical-pathologic features of tumor, microenvironment, location, and metastasis (Diaz and Bardelli, 2014; Chen et al., 2019). These cfDNAs enter to the bloodstream *via* processes of apoptosis, necrosis, secretion, autophagy, and necroptosis (Thierry et al., 2016). Pathological cell death conditions such as necrosis, autophagy, or mitotic catastrophe in cancer conditions result in the release of smaller cfDNA fragments into the circulation as opposed to large cfDNA fragments in physiological apoptotic cell death (Giacona et al., 1998; Jin and El-Deiry, 2005). More fragmented cfDNA and its higher concentration have been reported in cancer patients of various aetiologies (Stroun et al., 1989; Fleischhacker and Schmidt, 2007). The cfDNA integrity index (cfDII) is the calculated as the ratio of large to small DNA fragment concentrations of a known gene to measures cfDNA fragmentation. The smaller fragments are <180bp size which corresponds to apoptotic DNA fragmentation size. The selection of genes for cfDII are mostly repetitive DNA elements as there is high probability of it to be released into circulation or specific housekeeping genes to be used as cancer biomarker (Stroun et al., 2001). Increased cfDII of ALU (Arthrobacter luteus) elements was reported in endometrial cancer (Vizza et al., 2018), colorectal (Umetani et al., 2006a), breast (Tang et al., 2018), and prostate cancer (Khani et al., 2019). The cfDII of other repeat elements LINE-1 and housekeeping genes such as β-actin and GAPDH are also explored as liquid biopsy marker in different cancers including HCC (Chen et al., 2012), pancreatic cancer (Tuchalska-Czuron et al., 2020), breast cancer (Cheng et al., 2018), and renal cell carcinoma (Gang et al., 2010). The cfDII was also studied to differentiate breast cancer (Umetani et al., 2006b) or HCC (Wu et al., 2020) from healthy subjects. However, both increased and decreased cfDII levels are reported in cancer, depending upon the absolute and relative quantification methods of real-time quantitative PCR (RT-qPCR) (Madhavan et al., 2014; Cheng et al., 2017). To avoid confusion of cfDII interpretations and to use as a biomarker, we renamed it as cfDII-integrity and cfDII-fragmentation. The cfDII-integrity is the ratio of large to small DNA fragment (L/S ratio) concentration, which is determined by absolute quantification method of RT-qPCR that determine mainly the integrity of cfDNA. Higher integrity is expected in healthy individuals as the L/S ratio is closer to 1, whereas, decreased cfDII-integrity is expected in cancer. In the relative quantification method, normalization with a calibrator using either healthy control cfDNA or genomic DNA is used. The cfDII determined by this method estimates smaller fragment concentrations with reference to larger fragment concentrations in the same individual. It takes care of variations of large to small fragment concentrations in the same individuals and accurately measure of cfDNA fragmentation hence renamed as cfDII-fragmentation. This is expected to rise in log fold in cancer patients; hence, it can be used as a better liquid biopsy marker for cancer or HCC progression from CLD.

In this study, we used RT-qPCR to determine the cfDII by absolute and relative quantification methods. Both cfDII-integrity and cfDII-fragmentation was evaluated for four genes including repetitive elements (ALU and LINE-1) and housekeeping (GAPDH and β-actin) genes to differentiate HCC from CLD patients by plotting receiver operating characteristic (ROC) curve.

## 2 Materials and methods

### 2.1 Patients

This prospective observational study was carried out at the All India Institute of Medical Sciences, New Delhi, India, a tertiary care hospital, from February 2019 to July 2021. The study protocol was approved by the institute’s ethical committee (Reference number: IECPG-38/23.01.2019, RT-13/28.02.2019). A total of 200 consecutive patients (HCC, n = 100 and CLD, n = 100) attending the liver clinics were included in this study. The male and female proportions in HCC were 84% and 16%, and in the CLD patient population, the proportions were 67% and 33% respectively. HCC was diagnosed as per the European Association for the Study of the Liver (EASL) criteria (EASL, 2012; Galle et al., 2018), and both viral (HBV, HCV) and non-viral (alcoholic and non-alcoholic) etiologies were included. All HCC patients were staged as per the BCLC classification (Llovet et al., 1999; Bruix and Sherman, 2011), and all BCLC stage (A–D) patients were included. Similarly, all consecutive chronic liver disease (CLD) patients, including cirrhotic and non-cirrhotic patients of viral (CHB and CHC) and non-viral etiologies, were included. All participants were more than 18 years old and gave written consent for this study. Those with HIV, pregnant women, renal failure, and sepsis were excluded from the study. Healthy volunteers (n = 30) negative for HBV and HCV were included as controls. The demographic profile and clinical and biochemical parameters of all patients were recorded.

### 2.2 Blood sample collection and cell-free DNA isolation

The blood samples were collected from patients at one time point before start of anticancer treatment. The peripheral blood samples were collected in a vacutainer with a gel clot activator and quickly serum was separated to avoid damage to the genomic DNA. The cfDNAs were isolated from serum samples by the QIAsymphony automated nucleic acid extraction system (Qiagen) using the QIAamp DSP (Diagnostic Sample Preparation) mini nucleic acid isolation kit (Qiagen) as per the manufacturer’s protocol. Briefly, 400 μL of serum was used as starting material, and the elution volume was 40 μL. The concentration of cfDNA was measured using a Multi-ScanGo spectrophotometer (Thermo Fischer), and the cfDNA purity was analysed using the A260/A280 ratio.

### 2.3 Genomic DNA isolation and PCR amplification of ALU, LINE-1, GAPDH, and β-actin gene small and large fragments

**FIGURE 1:**
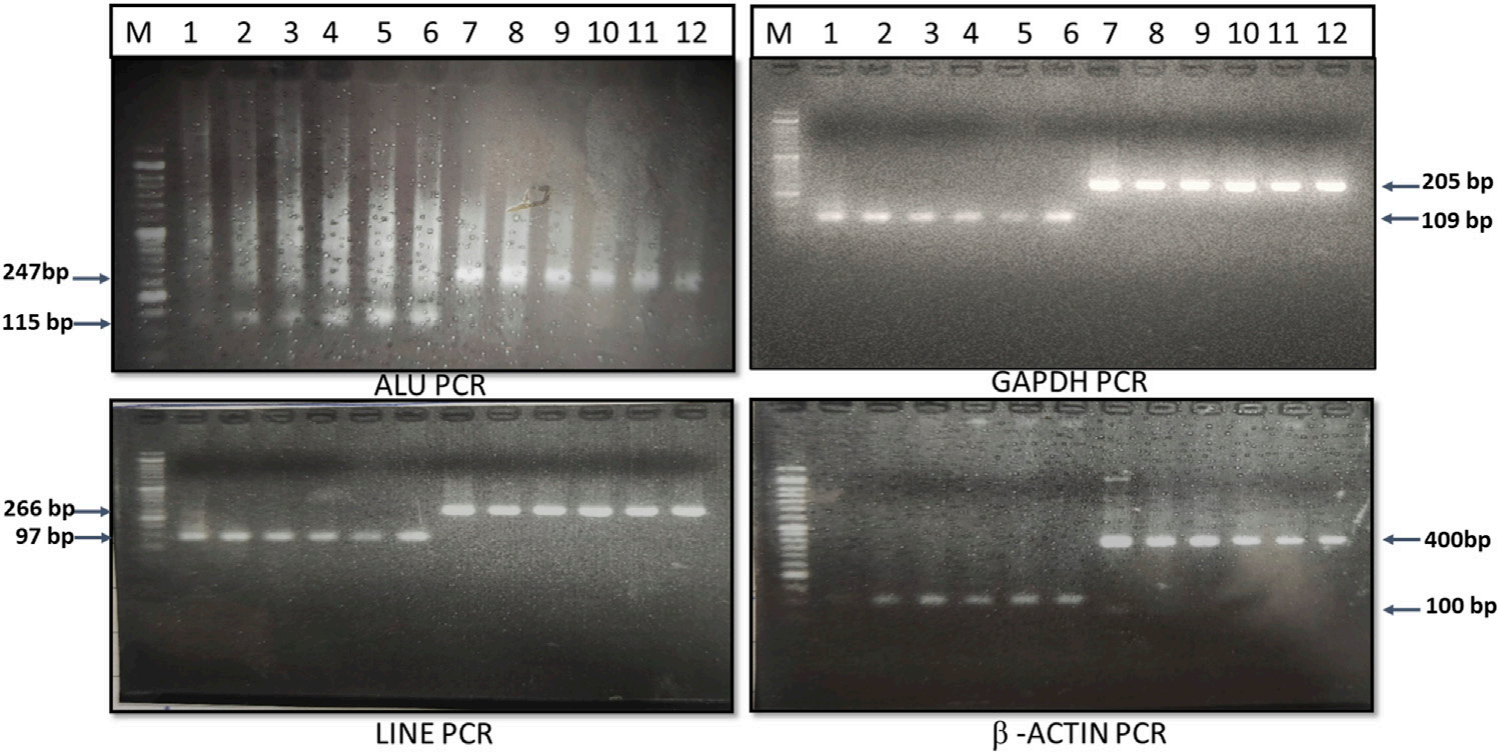
PCR amplification and gel electrophoresis of ALU, LINE, GAPDH and β-actin gene.

The genomic DNAs from whole blood was isolated using the QIAamp mini genomic DNA isolation kit (Qiagen), and the genomic DNA from the Huh-7 hepatoma cell line was isolated using the Proteinase-K digestion, phenol-chloroform extraction, and ethanol precipitation method. These genomic DNAs were used as a template for amplification of small and large fragments of ALU (115 bp and 247 bp), LINE-1 (97 bp and 266 bp), β-actin (100 bp and 400 bp), and GAPDH (110 bp and 205 bp) genes. The primers for amplification of ALU, LINE-1, β-actin and GAPDH gene small and large fragments were custom designed using NCBI reference sequence, verified with previously published primer sequence (Sobhani et al., 2018) and mentioned in Table 1. The desired fragment size was confirmed by 2% agarose gel electrophoresis (Figure 1).

**TABLE 1:** Primers for amplification of large and small fragments of ALU, LINE-1, GAPDH and β-actin gene.

Gene	Primer sequence (5’ →3′)	Amplicon size
ALU	CCT​GAG​GTC​AGG​AGT​TCG​AG (Forward)	Small (115 bp)
	CCC​GAG​TAG​CTG​GGA​TTA​CA (Reverse)	
	GTG​GCT​CAC​GCC​TGT​AAT​C (Forward)	Large (247 bp)
	CAGGCTGGAGTGCAGTGG (Reverse)	
LINE-1	TGG​CAC​ATA​TAC​ACC​ATG​GAA (Forward)	Small (97 bp)
	TGA​GAA​TGA​TGG​TTT​CCA​ATT​TC (Reverse)	
	ACT​TGG​AAC​CAA​CCC​AAA​TG (Forward)	Large (266 bp)
	CAC​CAC​AGT​CCC​CAG​AGT​G (Reverse)	
β-Actin	GCA​CCA​CAC​CTT​CTA​CAA​TGA (Forward)’	Small (100 bp)
	GTC​ATC​TTC​TCG​CGG​TTG​GC (Reverse)	
	GCA​CCA​CAC​CTT​CTA​CAA​TGA (Forward)	Large (400 bp)
	TGTCACGCACGATTTCCC (Reverse)	
GAPDH	TGG​CAC​ATA​TAC​ACC​ATG​GAA (Forward)	Small (110 bp)
	TGA​GAA​TGA​TGG​TTT​CCA​ATT​TC (Reverse)	
	GGATTTGGTCGTATTGGG (Forward)	Large (205 bp)
	GGA​AGA​TGG​TGA​TGG​GAT​T (Reverse)	

### 2.4 Concentration of cfDNA small and large fragments by real-time quantitative PCR of ALU, LINE-1, GAPDH, and β-actin gene

Real-time qPCR for ALU, LINE-1, β-actin, and GAPDH genes using SYBR green chemistry was used to determine the concentration of large (>205 bp) and small (110 bp) fragments of cfDNA in the sera of healthy, CLD, and HCC patients. RT-qPCR was carried out in a 10 µL final reaction volume using the Rotor-Gene Q real-time PCR (Qiagen) System. Each reaction mixture contained 2 µL (∼10 ng) of cfDNA template, 0.2 µL forward and reverse primer (10 µM), 5 µL 2X SYBR green (Agilent), and 2.4 µL nuclease-free water (Qiagen). The reaction condition was 95°C for 5s (seconds), followed by 40 cycles of 95°C for 15 s, annealing (58°C, 52°C, 55°C, and the 50°C) for 20s, and extension at 72°C for 30s. The Ct value of the RT-qPCR test was used to determine the absolute concentration of fragments by interpolating it from the genomic DNA standard curve of known DNA concentrations ranging from 20 ng to 0.02 pg. The concentration of smaller fragments relative to larger fragments was determined by the comparative Ct (2 ^–ΔΔCt^) method. Normalization using the mean Ct value of Huh-7 cell genomic DNA was carried out. Assuming a small fragment as target and a large fragment as a calibrator, normalization was carried out using a reference Ct value of Huh-7 cell genomic DNA or healthy control cfDNA.

### 2.5 DNA integrity index and total cfDNA concentration

The cfDII is derived from the ratio of large-to-small (L/S) DNA fragments and named as L/S ratio or cfDII-integrity. It was measured for ALU 247/115, LINE 266/97, β-actin 400/100 and GAPDH 205/110 genes in healthy, CLD and HCC patients. The cfDII was derived using the absolute concentration of large to small fragments as described earlier (Umetani et al., 2006b). The absolute concentration of small fragments is indicative of total serum cfDNA concentration. The GAPDH gene small (110 bp) fragment concentration was measured to compare cfDNA concentration in healthy, CLD, and HCC patients. An L/S ratio or cfDII-integrity closer to 1 is indicative of better DNA integrity using the absolute quantitation method, which was determined for all four genes in healthy, CLD, and HCC patients.

The cfDII determined by comparative Ct method (Wang et al., 2003) as per formula DII = exponential of (-ΔΔCt * ln2) or e^[-ΔΔCt * ln2 (0.693)] or 2^^(−ΔΔCt)^ is named as cfDII-fragmentation. The cfDII-fragmentation measures the true level of fragmented cfDNA (smaller fragment) by subtracting large fragment from total cfDNA. Normalization using Huh-7 cell genomic DNA mean Ct value and healthy control cfDNA mean Ct value was carried out for comparison of cfDII-fragmentation among healthy, CLD, and HCC patients.

### 2.6 Statistical analysis

Statistical analysis was performed using GraphPad Prism 9.4.1 and SPSS software. The clinical, demographic profile data of patients expressed as mean ± standard deviation. All the parametric data among groups were compared using ANOVA and between two groups were compared by *t*-test. The cfDII-fragmentation and cfDII-integrity in different groups were non-parametric and expressed as a median (IQR). These non-parametric data among healthy, CLD and HCC groups were compared using Kruskal–Wallis test followed by Dunns multiple comparison test for posthoc analysis. The cfDII-fragmentation (normalization with healthy control cfDNA) of CLD and HCC groups as non-parametric data were compared by Mann-Whitney *U* Test. The pool sample size was calculated considering HCC patients as cases and CLD patients as controls using Alu-cfDII as the quantitative variable to determine differences in mean (d) and standard deviation (SD). Assuming 80% power (Z_β_), 5% level of significance (Z_α/2_), and case: control ratio (r) = 1, the required sample size was calculated to be 79 for both case and controls using the formula: (r+1/r)[SD^2^ *(Z_α/2_ +Z_β_)^2^/d^2^). To evaluate the diagnostic utility of the cfDNA integrity index (DII), the area under the receiver-operating characteristic (AUROC) curve was plotted. The sensitivity, specificity, and accuracy were calculated accordingly using the cut-off value when the Youden index was maximal. All *p* values were two-sided, and *p* < 0.05 was considered statistically significant.

## 3 Results

### 3.1 Demographic, clinical, and biochemical profiles of the study population

HCC patients had a higher mean age (54 ± 13.14 years, n = 100) than CLD patients (36.08 ± 14.22 years, n = 100), with a significant male predominance in both cases (CLD, 67% and HCC, 84%). The major etiologies for both cases were viral infection (CLD, 87% and HCC, 61%) followed by chronic alcohol consumption (CLD, 6%and HCC, 16%). The percentages of liver cirrhosis in CLD and HCC patients were 32% and 89%, respectively. Other biochemical and clinical parameters were mentioned in Table 2.

**TABLE 2:** Demographic, biochemical and clinical profile of study population and comparison between HCC, CLD and Healthy cohorts.

Sr. No	Variable	Healthy (n = 30)	CLD (n = 100)	HCC (n = 100)	*p* value
1	Age (Year, Mean ± SD)	29.62 ± 7.63	36.08 ± 14.22	54 ± 13.14	**0.001**
2	Sex (n, %)	8 (27%)	67 (67%)	84 (84%)	**0.008**
	Male	22 (73%)	33 (33%)	16 (16%)	
	Female				
3	Etiologies (n, %)	-	65 (65%)	45 (45%)	**0.004**
	HBV	-	22 (22%)	16 (16%)	0.28
	HCV	-	6 (6%)	8 (8%)	0.58
	Alcohol	-	-	6 (6%)	----
	HBV + Alcohol	-	-	2 (2%)	-----
	HCV + Alcohol	-	2 (2%)	5 (5%)	0.25
	HVOTO	-	5 (5%)	11 (11%)	0.11
	NASH	-	-	3 (3%)	----
	HBV + HCV	-	-	4 (4%)	-----
	Cryptogenic				
4	Child’s Score	-	-	88 (88%)	NA
	A	-	-	11 (11%)	
	B	-	-	1 (1%)	
	C				
5	BCLC Stage	-	-	45 (45%)	NA
	A	-	-	38 (38%)	
	B	-	-	15 (15%)	
	C	-	-	2 (2%)	
	D				
6	PST Score	-	-	71 (71%)	NA
	0	-	-	24 (24%)	
	1	-	-	03 (3%)	
	2	-	-	02 (2%)	
	3				
7	Type II Diabetes Mellitus (n, %)	-	8 (8%)	21 (21%)	0.07
8	Cirrhosis	-	32 (32%)	89 (89%)	**0.001**
9	AFP (ng/ml)	-	-	14,835.2 ± 113,567.5	NA
	<20 (ng/ml)	-	-	22 (22%)	
	>20 (ng/ml)			78 (78%)	
10	Serum Albumin (g/dl)	-	4.45 ± 0.68	4.25 ± 4.52	0.64
11	Bilirubin (mg/dl)	-	0.85 ± 0.59	1.34 ± 1.49	**0.0025**
12	AST (IU/ml)	-	81.75 ± 63.78	76.46 ± 83.03	0.61
13	ALT (IU/ml)	-	83.70 ± 104.88	60.91 ± 83.98	0.08
14	SAP (IU/ml)	-	234.23 ± 112	316.96 ± 321.62	**0.016**
15	Total Protein (g/dl)	-	7.5 ± 0.49	7.4 ± 0.61	0.20

All values are expressed as n (%) or (mean ± SD) unless otherwise specified. *p*-value obtained from statistical analysis shows comparison between HCC, CLD, and Healthy subjects.

Abbreviations: AST, Aspartate aminotransferase; ALT, Alanine aminotransferase; SAP, Serum alkaline phosphatase; PT, Prothrombin time; Hb, Hemoglobin; TLC, Total leucocyte count; PLT, Platelet count; AFP, Alpha-feto protein; HBV, Hepatitis B virus; HCV, Hepatitis C virus: NASH, Non-alcoholic steatosis Hepatitis; HVOTO, Hepatic venous outflow tract obstruction.

Bold values are statistically significant (*p*<0.05).

### 3.2 Total cfDNA concentration in healthy, CLD, and HCC patients

Total cfDNA concentration in the serum of healthy controls, CLD, and HCC patients was assessed using qRT-PCR of GAPDH’s smaller (110 bp) fragment. The abundance of repetitive elements (ALU and LINE-1) and β-actin gene concentration as compared to GAPDH was determined using the large fragment concentration of genomic DNA. The ratio of β-actin, LINE1 and ALU to GAPDH concentrations was 0.05: 2500: 5833. In the healthy control, the median cfDNA total concentration in the sera was 16.88 ng/ml (IQR: 1.66–62.59 ng/ml). Total cfDNA concentrations in CLD and HCC patients were 33 ng/ml (IQR: 0.21–105 ng/ml) and 244 ng/ml (IQR: 179–287 ng/ml), respectively. The total cfDNA concentration in HCC patients was significantly higher than in healthy and chronic liver disease controls (*p* = 0.0001).

### 3.3 The cfDII-integrity or L/S ratio as liquid biopsy marker to differentiate HCC from healthy and CLD patients

**FIGURE 2:**
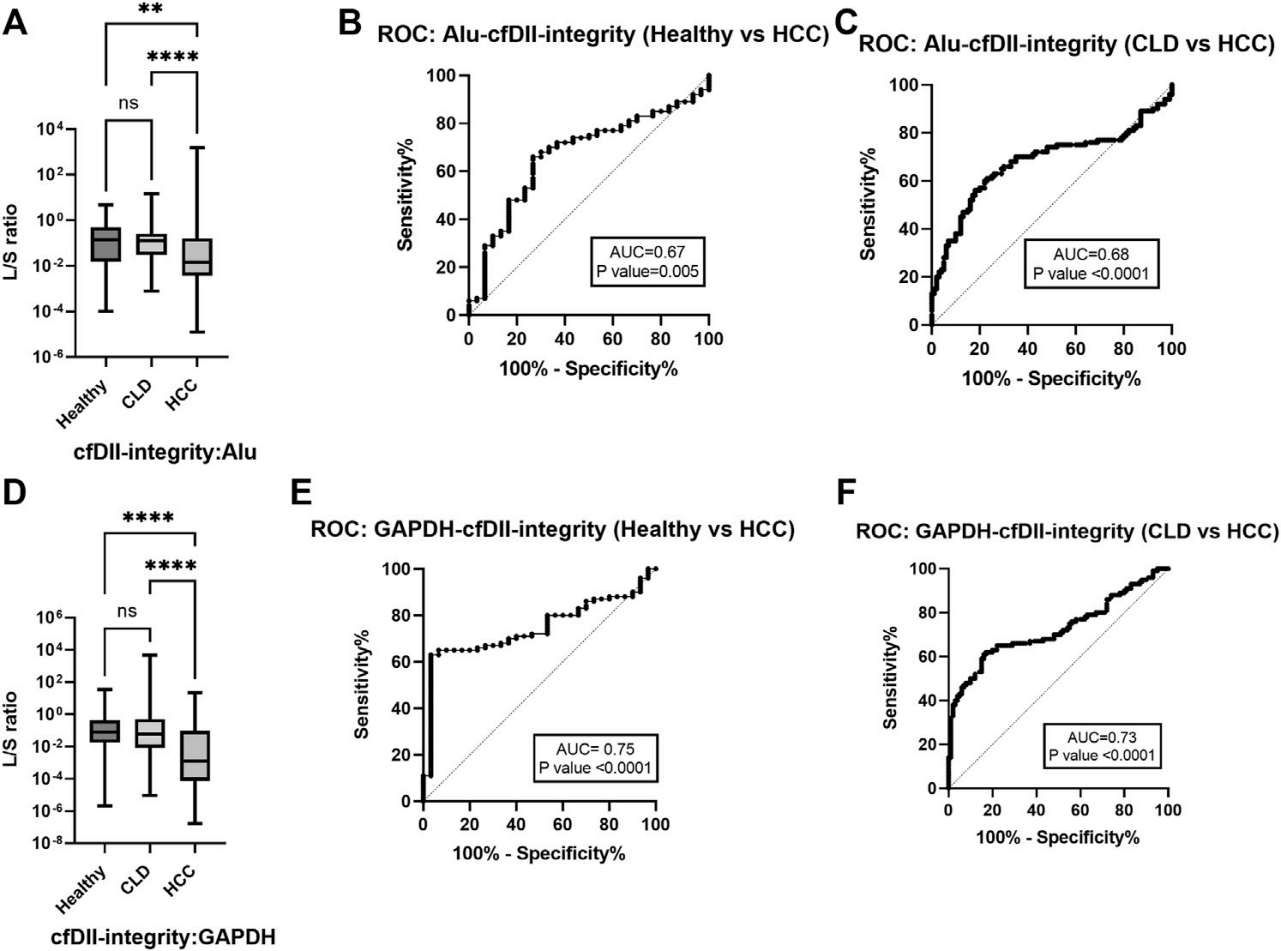
The cfDII-integrity (L/S ratio) differentiating HCC from CLD and healthy subjects. **(A)** ALU gene cfDII-integrity (L/S ratio) among healthy, CLD and HCC patients; **(B)** The ROC plot of ALU gene cfDII-integrity differentiating HCC from healthy subjects; **(C)** The ROC plot of ALU gene cfDII-integrity differentiating HCC from CLD patients, **(D)** GAPDH gene cfDII-integrity (L/S ratio) among healthy, CLD and HCC groups; **(E)** The ROC plot of GAPDH gene cfDII-integrity differentiating HCC from healthy subjects; **(F)** The ROC plot of GAPDH gene cfDII-integrity differentiating HCC from CLD patients, ns- Non significant, **- *p* value = 0.005, ****- *p* value < 0.0001.

The ratio of large to small DNA fragment concentration (L/S ratio) as a measure of cfDII-integrity was determined by absolute quantification of RT-qPCR and mentioned in Table 3A for ALU, LINE-1, GAPDH and β-actin. The median value of ALU gene cfDII-integrity was found significant (*p* < 0.0001) in Kruskal–Wallis test among groups: healthy (0.1408), CLD (0.1275) and HCC (0.0145) patients. In post hoc analysis, the cfDII-integrity of HCC vs. healthy (*p* = 0.0051), HCC vs. CLD (*p* < 0.0001) was found significant (Figure 2A). The AUROC differentiating healthy and CLD from HCC was found significant. The AUROC for healthy vs. HCC was 0.67, *p* = 0.005, and the cut-off point is 0.05 at 68% sensitivity and 70% specificity (Figure 2B). The AUROC for CLD vs. HCC was 0.67, *p* < 0.0001 (Figure 2C) with the cut off value <0.05 at 68% sensitivity and 67% specificity.

**TABLE 3:** Comparison of ALU, LINE-1, GAPDH and β-actin gene cfDII-integrity and cfDII-fragmentation among healthy, CLD and HCC patients.

Gene	Healthy (Llovet et al., 2021)	CLD (Sung et al., 2021)	HCC (Dikshit et al., 2012)	*p*-value	Posthoc, *p* value
**(A) The cfDII-integrity (L/S ratio): Absolute quantification, median (IQR) value**				**Kruskal–wallis test**	**Dunns multiple comparison test**
ALU	0.141 (0.015–0.501)	0.127 (0.029–0.256)	0.014 (0.003–0.163)	**<0.0001**	1 vs. 2, 1.0
					**1 vs. 3, 0.0051**
					**2 vs. 3, <0.0001**
LINE-1	0.022 (0.001–0.204)	0.025 (0.005–0.058)	0.022 (0.006–0.103)	0.809	1 vs. 2, >0.9999
					1 vs. 3, 0.8653
					2 vs. 3, >0.9999
GAPDH	0.078 (0.017–0.429)	0.058 (0.008–0.523)	0.001 (0.00007–0.093)	**0.0001**	1 vs. 2, 1.0
					**1 vs. 3, <0.0001**
					**2 vs. 3, <0.0001**
β-Actin	0.210 (0.031–0.453)	0.066 (0.016–0.220)	0.093 (0.032–1.000)	**0.042**	1 vs. 2, 0.1774
					1 vs. 3, 1.0
					2 vs. 3, 0.0822
**(B) The cfDII-fragmentation normalization with gDNA: Relative quantification, median (IQR) value**				**Kruskal–Wallis test**	**Dunns Multiple comparison Test**
ALU	2.334 (0.815–14.446)	2.522 (1.414–8.430)	15.189 (2.067–47.752)	**<0.0001**	1 vs. 2, 1.0
					**1 vs. 3, 0.005**
					**2 vs. 3, <0.0001**
LINE-1	2.930 (0.310–53.629)	2.808 (1.208–12.104)	3.295 (0.686–11.196)	0.827	1 vs. 2, 1.0
					1 vs. 3, 1.0
					2 vs. 3, 1.0
GAPDH	7.412 (1.942–24.199)	6.233 (1.268–27.57)	64.222 (1.855–831.366)	**<0.0001**	1 vs. 2, 1.0
					**1 vs. 3, 0.01**
					**2 vs. 3, <0.0001**
β-Actin	1.607 (0.817–8.477)	4.423 (1.539–14.898)	3.271 (0.406–8.414)	**0.034**	1 vs. 2, 0.13
					1 vs. 3, 1.0
					2 vs. 3, 0.07
**(C) The cfDII-fragmentation normalization with healthy control cfDNA: Relative quantification, median (IQR) value**				**Mann-Whitney U Test**	
ALU		0.729 (0.408–2.436)	4.389 (0.597–13.799)	**<0.0001**	
LINE-1		0.566 (0.243–2.441)	0.664 (0.138–2.257	0.531	
GAPDH		1.248 (0.228–5.856)	13.784 (0.398–178.445)	**<0.0001**	
β-Actin		1.586 (0.551–5.341)	1.173 (0.145–3.016)	0.086	

Statistical significance is the *p* value < 0.05 and significant values are mentioned in bold font.

The median value of GAPDH gene cfDII-integrity was found significant (*p* = 0.0001) in Kruskal–Wallis test among groups: healthy (0.078), CLD (0.058) and HCC (0.001) patients. In post hoc analysis, the cfDII-integrity of HCC vs. healthy (*p* < 0.0001), HCC vs. CLD (*p* < 0.0001) was found significant (Figure 2D). The GAPDH gene cfDII-integrity AUROC was 0.7453, *p* < 0.000, which can differentiate healthy vs. HCC and the cut-off point is <0.02 at 67% sensitivity and 73.3% specificity (Figure 2E). The AUROC for CLD vs. HCC was 0.7257, *p* < 0.0001 (Figure 2F), with the cut off value <0.02 at 66% sensitivity and specificity. The cfDII-integrity of LINE-1 and β-actin gene was not found significant (Table 3A).

### 3.4 The cfDII-fragmentation as liquid biopsy marker to differentiate HCC from healthy and CLD patients following normalization with cellular genomic DNA

**FIGURE 3:**
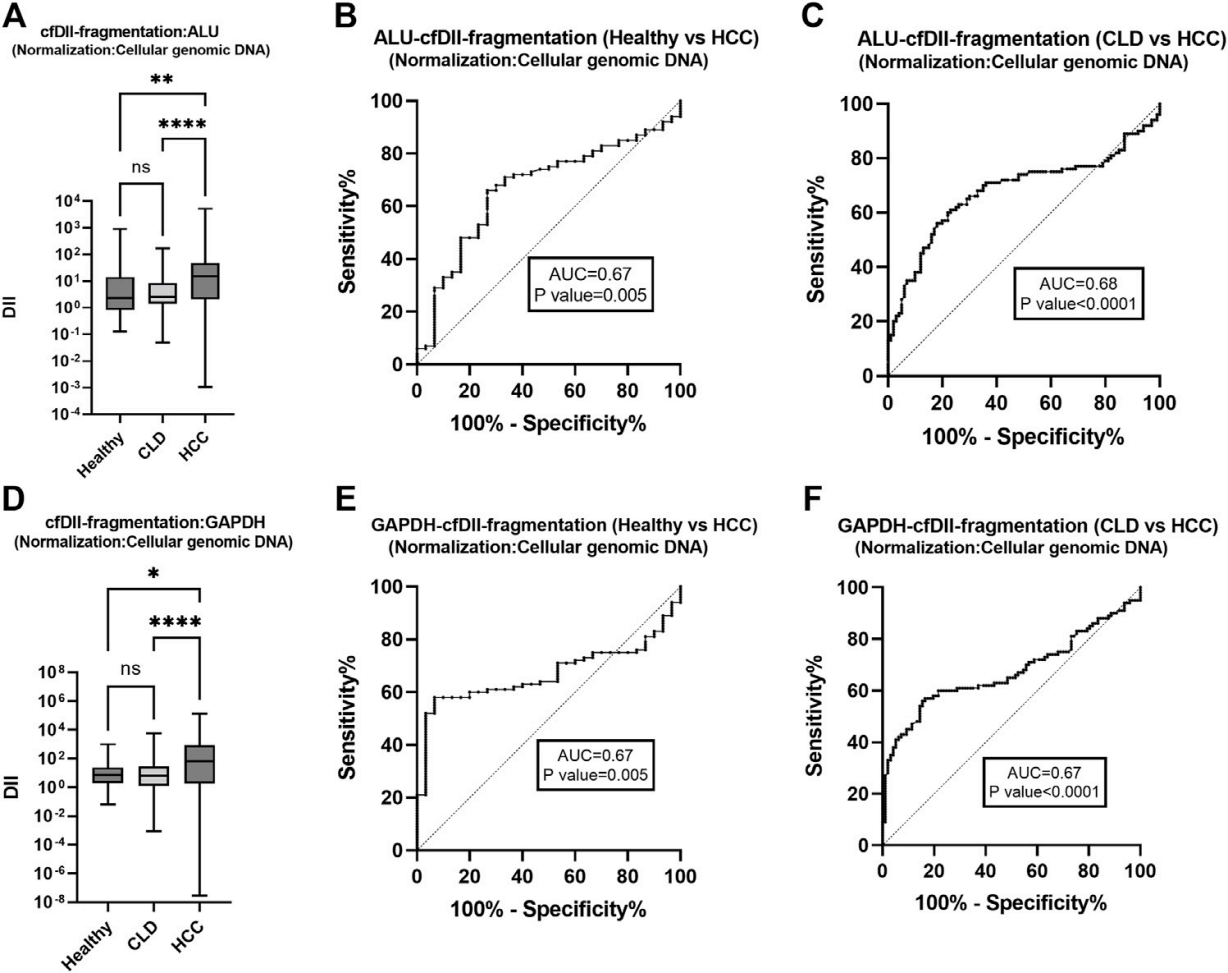
The cfDII-fragmentation following normalization with cellular genomic DNA for comparison among healthy, CLD and HCC patients. The ALU gene cfDII-fragmentation among healthy, CLD and HCC patients following normalization with cellular genomic DNA **(A)**. The ROC plot of ALU gene cfDII-fragmentation differentiating HCC from healthy subjects **(B)**, HCC from CLD patients **(C)**. The GAPDH gene cfDII-fragmentation among healthy, CLD and HCC patients following normalization with cellular genomic DNA **(D)**. The ROC plot of ALU gene cfDII-fragmentation differentiating HCC from healthy subjects **(E)**, HCC from CLD patients **(F)**. ns- Non significant, *- *p* value = 0.01, **- *p* value = 0.005, ****- *p* value < 0.0001.

The cfDII-fragmentation which determine fragmentation of cfDNA was calculated among healthy, CLD, and HCC patient groups following normalization with Huh-7 cell genomic DNA. The cfDII-fragmentation as a measure of cfDNA fragmentation was determined by relative quantification method using RT-qPCR. The cfDII-fragmentation median and IQR value for ALU, LINE-1, GAPDH and β-actin genes were mentioned in Table 3B. The median value of ALU gene cfDII-fragmentation was found significant (*p* < 0.0001) in Kruskal–Wallis test among groups: healthy (2.334), CLD (2.522) and HCC (15.189) patients. In post hoc analysis, the cfDII-fragmentation was found significant for HCC vs. healthy (*p* = 0.005), HCC vs. CLD (*p* < 0.0001) (Figure 3A). The AUROC for healthy vs. HCC patients cfDII-fragmentation was 0.67 and found significant *p* < 0.0001 with cut-off value >5.4 at 68% sensitivity and 70% specificity (Figure 3B). The AUROC for CLD vs. HCC patients cfDII-fragmentation was 0.68, *p* < 0.0001 with the cut off value 5.5 at 68% sensitivity and 67% specificity (Figure 3C).

The median value of GAPDH gene cfDII-fragmentation was found significant (*p* < 0.0001) in Kruskal–Wallis test among groups: healthy (7.412), CLD (6.233) and HCC (64.222) patients. In post hoc analysis, the cfDII-fragmentation was found significant for HCC vs. healthy (*p* = 0.01) and HCC vs. CLD (*p* < 0.0001) (Table 3B and Figure 3D). The AUROC of GAPDH gene cfDII-fragmentation was 0.67, *p* = 0.004, which can differentiate healthy vs. HCC with a cut-off value of >10.14 and a sensitivity and specificity of 62% (Figure 3E). The AUROC of GAPDH gene cfDII-fragmentation for CLD vs. HCC was 0.674, *p* < 0.0001 with a cut off value of 11.08 at 62% sensitivity and specificity (Figure 3F). The cfDII-fragmentation of LINE-1 and β-actin gene was not found significant (Table 3B).

### 3.5 The cfDII-fragmentation as liquid biopsy marker to differentiate HCC from CLD patients following normalization with healthy control cfDNA

**FIGURE 4:**
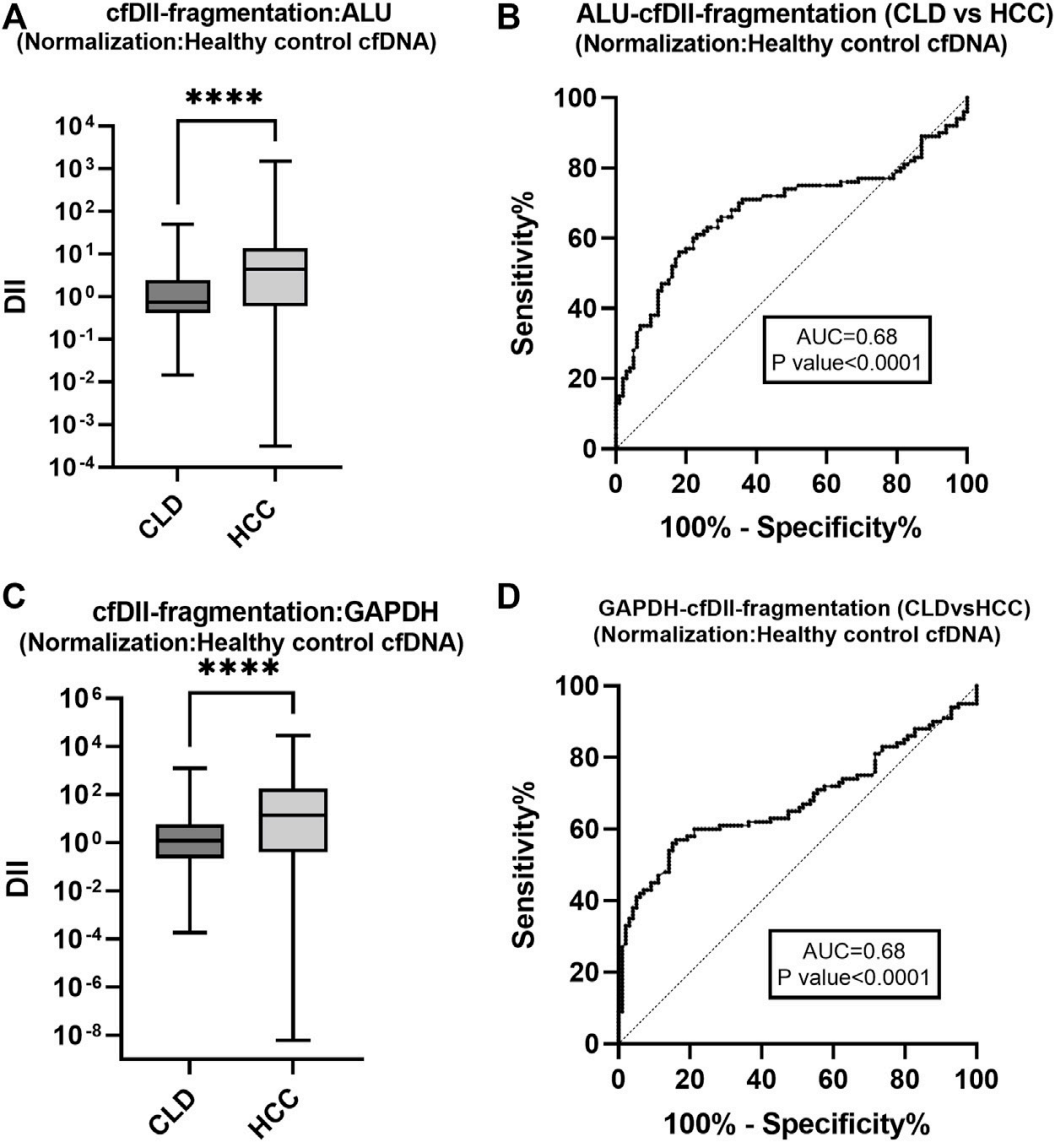
The cfDII-fragmentation following normalization with healthy control cfDNA for comparison between CLD and HCC patients. The cfDII-fragmentation median value of CLD and HCC patients following normalization with healthy control cfDNA for ALU element **(A)** and GAPDH gene **(C)**. The ROC plot of ALU element cfDII-fragmentation differentiating HCC from CLD patients **(B)**. The GAPDH gene cfDII-fragmentation ROC plot differentiating HCC from CLD patients **(D)**. There is an obvious similarity observed between Figure 4B with Figure 3C for ALU element and between Figure 4D with Figure 3F for GAPDH gene. Both of these genes cfDII-fragmentation differentiate HCC from CLD. The primary data are the same for both genes, only the normalization method is different one is with cellular genomic DNA (Figures 3C,F) other is with healthy control cfDNA (Figures 4B,D). Therefore, the ROC pattern is similar even though data and cut-off points are different after normalization. ****- *p* value < 0.001.

The cfDII-fragmentation following normalization with healthy control cfDNA was calculated by relative quantification method using RT-qPCR for comparison between two groups (CLD and HCC). The cfDII-fragmentation of ALU, LINE-1, GAPDH and β-actin genes median and IQR value were mentioned in Table 3C. The non-parametric data between two groups were compared using Mann-Whitney test and found significant (*p* < 0.0001) for ALU (Figure 4A) and GAPDH (Figure 4C) genes (Table 3C). The ALU gene cfDII-fragmentation of HCC patients (4.389, IQR-0.60–13.83) was significantly (*p* < 0.0001) higher than that of CLD (0.729, IQR-0.40–2.4) patients (Table 3C; Figure 4A). The AUROC that distinguished HCC from CLD was 0.68 (*p* = 0.0001) and the cut off value of cfDII-fragmentation > 2.03 with 65.6% sensitivity and 71% specificity (Figure 4B).

The GAPDH gene cfDII-fragmentation following normalization with healthy control cfDNA found significantly (*p* < 0.0001) higher in HCC patients (13.784, IQR-0.398–178.44) than that of CLD (1.248, IQR-0.228–5.856) patients (Table 3C; Figure 4C). The AUROC for CLD vs. HCC was 0.67, *p* < 0.0001 (Figure 4D). The cut-off value for distinguishing CLD from HCC was found to be 2.37, with 62.6% sensitivity and 63.64% specificity. The cfDII-fragmentations of LINE-1 and β-actin genes for CLD and HCC patients were not found significant (Table 3C).

### 3.6 The cfDII-fragmentation trends in different etiologies of HCC

**FIGURE 5:**
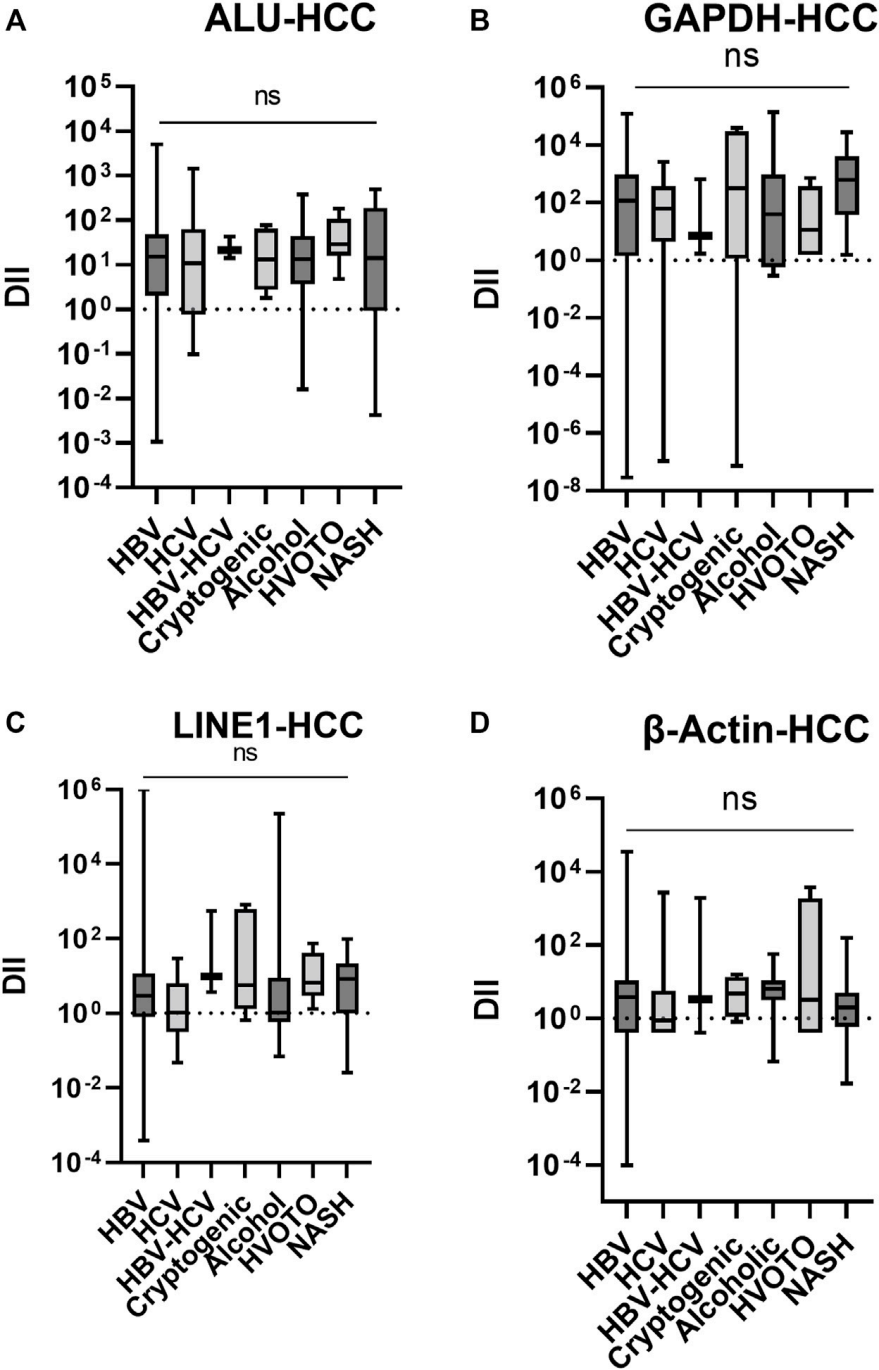
The cfDII-fragmentation trends in HCC patients following normalization with healthy control cfDNA for ALU **(A)**, LINE **(B)**, GAPDH **(C)** and β-actin **(D)** gene. ns, non significant.

The cfDII-fragmentations following normalization with healthy control cfDNA were further evaluated in HCC patients with various etiologies, including viral and non-viral. In Kruskal–Wallis test, the data were not found significant and only trends were depicted in Figure 5. The median cfDII-fragmentation of ALU elements (Figure 5A) were HBV,15.14; HCV, 10.81; HBV + HCV, 21.41; Cryptogenic, 13.19; Alcohol, 13.36; HVOTO, 29.04, NASH, 13.98. The median cfDII of LINE1 elements (Figure 5B) were HBV, 2.928; HCV, 1.057; HBV + HCV, 9.448; Cryptogenic, 5.697; Alcohol, 1.05; HVOTO, 6.589, NASH, 8.15. Higher cfDII-fragmentation was observed for ALU than LINE elements for HCC of different etiologies.

The median cfDII-fragmentation of GAPDH gene (Figure 5C) in HCC patients of different etiologies were HBV, 117.0; HCV, 61.67; HBV + HCV, 6.916; Cryptogenic, 313.1; Alcohol, 39.12; HVOTO, 11.16, NASH, 611.1. The median cfDII-fragmentation of -actin gene in HCC (Figure 5D) patients were HBV, 3.793; HCV, 0.8594; HBV + HCV, 3.317; Cryptogenic, 4.653; Alcohol, 6.277; HVOTO, 3.182, NASH, 1.974. For all etiologies of HCC, GAPDH had a higher cfDII-fragmentation value than β-actin gene.

## 4 Discussion

Chronic liver diseases that occur due to HBV and HCV infections, alcohol consumption, non-alcoholic fatty liver disease (NAFLD), autoimmune hepatitis, and hemochromatosis are all strongly associated with cirrhosis, HCC, and an increased risk of mortality. These CLD patients require routine viral load estimation, treatment, and monitoring for HCC. The liquid biopsy markers such as cfDNA or ctDNA can be useful for HCC screening due to the ease of the RT-qPCR technique, cost-effectiveness, frequent mass sample screening, and early detection of cancer. In this study, we used RT-qPCR to evaluate cfDII for ALU, LINE-1, β-actin, and GAPDH gene in healthy, CLD, and HCC patients as measures of cell-free DNA integrity (cfDII-integrity or L/S ratio) by absolute quantification methods and as measures of cell-free DNA fragmentation (cfDII-fragmentation) by relative quantification methods. We observed low cfDII-integrity (L/S ratio) and high cfDII-fragmentation in HCC patients as compared to CLD and healthy subjects. Using the ROC curve of both the cfDII-integrity and -fragmentation, we have shown that it can differentiate HCC from healthy individuals and CLD patients. Among the four selected genes, the RT-qPCR assay determining cfDII-integrity and -fragmentation for ALU and GAPDH have shown promising utility as an early biomarker for hepatocarcinogenesis by differentiating HCC from CLD.

Cell-free DNA is important for cancer diagnostics as a major portion of it is originated from the tumor itself resulting quantitative changes in the cfDNA concentration, integrity and fragmentation in the circulation of cancer patients (Schwarzenbach et al., 2011; Diaz and Bardelli, 2014). Total cfDNA concentration can be measured by spectrophotometry but the cfDNA concentration can be easily biased by pre-analytical techniques. Different cfDNA extraction kits/methods have different DNA recovery efficiencies (Page et al., 2013). Cell lysis may lead to increased cfDNA concentration (Page et al., 2006) whereas prolonged storage can decreases cfDNA concentration (Sozzi et al., 2005). The cfDNA concentration in healthy subjects ranges from 0 to 100 ng/ml of blood, with an average of 30 ng/ml whereas, the cfDNA concentration in cancer patients ranges from 0 to 1,000 ng/ml of blood, with an average of 180 ng/ml (Schwarzenbach et al., 2011). Using RT-qPCR of GAPDH gene, we have observed median cfDNA concentration of 16.88 ng/ml (IQR: 1.66–62.59 ng/ml) for healthy control, 33 ng/ml (IQR: 0.21–105 ng/ml) for CLD and 244 ng/ml (IQR: 179–287 ng/ml) for HCC patients. These results are consistent with previous findings of higher cfDNA DNA concentration in HCC patients than in healthy and CLD subjects (Diehl et al., 2005; Schwarzenbach et al., 2011).

The integrity of cfDNA is another parameter that changes in cancer patients. The cfDNA is more fragmented in cancer, with a higher prevalence of tumor-associated mutations in the shorter fragments (∼150–180 bp in length) of cfDNA in cancer patients (Diehl et al., 2005; Jiang et al., 2015). Larger fragments derived from apoptosis appear in multiples of ∼180-bp DNA fragments and are visible in gel as a DNA ladder, whereas necrotic DNA fragments are more non-specific and appear as a smear. DNA integrity is indicative of more cfDNA larger fragments in circulation due to apoptosis. Integrity of cfDNA expressed as a ratio of large to small fragments or DNA integrity index (DII), which is unaffected by external factors and accurately represents cancer-related DNA fragmentation. However, in cancer, both decreased (Madhavan et al., 2014; Cheng et al., 2017) and increased (Wang et al., 2003; Jiang et al., 2006; Leszinski et al., 2014) cfDII levels have been reported. This is primarily due to different approaches in determining DII. The DII determination was described in two ways: absolute quantification method using standard curve for small and large fragments concentration (L/S) ratio (Umetani et al., 2006b), and the relative quantification or comparative Ct method for large and small fragments (Wang et al., 2003). We have evaluated cfDII in both ways and plotted a ROC curve to differentiate HCC from healthy and CLD patients. The concentration of small fragments using RT-qPCR determines total cfDNA concentration and the L/S ratio closer to 1 indicates better integrity. We have observed cfDII-integrity (L/S ratio) closer to 1 in healthy and CLD subjects than in HCC subjects for ALU (Figure 2A) and GAPDH (Figure 2D), indicating better integrity. The cfDII-integrity was significantly able to differentiate HCC from healthy and CLD with AUROC of 0.67 at a cut-off of 0.05 for ALU elements (Figures 2B,C) and AUROC of 0.74 and 0.72, a cut-off of <0.02 for GAPDH gene (Figures 2E,F).

The other cfDII method proposed by Wang et al. (Wang et al., 2003) is the comparative ΔΔCt method, which involves normalizing the Ct value of small and large fragments by the healthy control or cultured cell genomic DNA mean Ct value to get ΔCt and then subtracting ΔCt of the large fragment from ΔCt of the small fragment to get ΔΔCt. Finally, DII was calculated to be as 2 ^−ΔΔCt^. This method yields a higher cfDII (Wang et al., 2003; Madhavan et al., 2014) and measures cfDNA fragmentation. We have observed increased cfDII-fragmentation in HCC as compared to healthy and CLD patients using Alu element (Figures 3A, 4B) and GAPDH (Figures 3D, 4C). We did not observe significant changes in cfDII-fragmentation of LINE1 and β-actin genes among healthy, CLD, and HCC patients (Table 3B). The cfDII-fragmentation of ALU and GAPDH can differentiate HCC from healthy and CLD patients. The AUROC of ALU element in distinguishing HCC from healthy (Figure 3B) and CLD (Figure 3C) is 0.67 and 0.68, with a cut-off value of >5.4 cfDII-fragmentation. Following normalization with healthy control, the AUROC differentiating HCC from CLD was 0.68, with a cut-off value of >2.03 (Figure 4B). Similarly, GAPDH cfDII-fragmentation differentiates HCC from healthy (AUROC, 0.67, Figure 3E) and CLD (AUROC, 0.67, Figure 3F) at cut-offs > 10.14 and >11.08, respectively. Following normalization with healthy control, the AUROC for GAPDH differentiating HCC from CLD was 0.67, with a cut-off value of >2.37 (Figure 4D). Increased levels of cfDNA in the serum or plasma of various HCC patient cohorts have been reported (Iizuka et al., 2006; Huang et al., 2012).

The increased cfDII-fragmentation in HCC is attributed to the cell necrosis within the tumour, resulting in an increased number of highly fragmented (smaller fragment) DNA copies released into circulation (Wang et al., 2003; Andersen et al., 2015). Increased cfDII-fragmentation was also observed in other cancer types, i.e., breast cancer, colorectal cancer, bladder cancer, and pancreatic cancer (Yu et al., 2014). Our results are consistent with previous studies. ALU cfDII-fragmentation has shown differentiating ability in colorectal cancer from healthy (Hao et al., 2014), -actin cfDII-fragmentation (394/99bp) has shown differentiating ability for breast cancer patients (Salimi and Sedaghati Burkhani, 2019). We did not observe any significant changes in -actin and LINE1 cfDII-fragmentation (Table 3). This may be attributed to cancer of different organs for β-actin whereas LINE1 mostly relates to the methylation patten of cfDNA. We have observed a similar pattern of cfDII-fragmentation in HCC patients of different aetiologies, which may indicate similar increased fragmentation of cfDNA in HCC regardless of its aetiology (Huang et al., 2016).

The limitations of our study include clinicopathological correlation of HCC with cfDII; changes in cfDII during hepatocarcinogenesis and disease progression; the effect of therapy on cfDII; and staging of HCC using cfDII. We did not perform any comparison of RT-qPCR based determination of cfDII with other liquid biopsy-based detections, such as: CTCs, CSCs, microRNAs, and exosomes in cancer. In conclusion, both cfDII-integrity and cfDII-fragmentation determined by RT-qPCR techniques for ALU elements and GAPDH gene can be useful biomarkers to differentiate HCC from CLD and healthy subjects. It has the potential to be an early hepatocarcinogenesis marker. Using cfDII to improve surveillance of CLD patients may help find more people with HCC at a curative stage.

## Data Availability

The original contributions presented in the study are included in the article/Supplementary Material, further inquiries can be directed to the corresponding author.
